# COVID-19 vaccination status during pregnancy and preeclampsia risk: the pandemic-era cohort of the INTERCOVID consortium

**DOI:** 10.1016/j.eclinm.2026.103785

**Published:** 2026-02-18

**Authors:** Paolo Ivo Cavoretto, Jose Villar, Antonio Farina, Marta Fabre, Philippe Deruelle, Agustin Conde-Agudelo, Adejumoke Idowu Ayede, Ernawati Ernawati, Constanza Soto Conti, Babagana Bako, Loïc Sentilhes, Satoru Ikenoue, Adele Winsey, Ken Takahashi, Shabina Ariff, Stephen Rauch, Gabriela Tavchioska, Michael Gravett, Ricardo Nieto, Federico Prefumo, Raffaele Napolitano, Francesco D'Ambrosi, Laurent J. Salomon, Anne Caroline Benski, Maria José Rodriguez-Sibaja, Roberto Casale, Sonia Deantoni, Nerea Maiz, Valeria Savasi, Irene Cetin, Manuela Oberto, Carmen Vecciarelli, Maria Carola Capelli, Becky Liu, Mohak Mhatre, Marynéa Silva do Vale, Saturday Etuk, Hadiza Shehu Galadanci, Jagjit S. Teji, Theresa Hubka, Helena Sobrero, Guadalupe Albornoz Crespo, Albertina Rego, Muhammad Baffah Aminu, Rachel Craik, Mustapha Ado Usman, Erkan Kalafat, Sarah Rae Easter, Vincent Bizor Nachinab, Eric Baafi, Mónica Savorani, Daniela Caceres, Perla K. García-May, Abimbola Bowale, Alexey Kholin, Leila Cheikh Ismail, Michal Lipschuetz, Carolina Giudice, Jim Thornton, Ramachandran Thiruvengadam, Sherief Abd-Elsalam, Eduardo A. Duro, Valeria Hernandez, Serena Gandino, Zulfi Bhutta, Brenda Eskenazi, Stephen Kennedy, Robert Gunier, Aris Papageorghiou

**Affiliations:** a Obstetrics and Gynaecology Department, IRCCS San Raffaele Hospital, Milan, Italy; b School of Medicine, University Vita Salute San Raffaele, Milan, Italy; c Nuffield Department of Women's & Reproductive Health, University of Oxford, Oxford, UK; d Oxford Maternal and Perinatal Health Institute, Green Templeton College, University of Oxford, Oxford, UK; e Obstetric Unit, IRCCS Azienda Ospedaliero-Universitaria di Bologna, Department of Medical and Surgical Sciences (DIMEC), Alma Mater Studiorum, University of Bologna, Bologna, Italy; f Department of Life, Health and Environmental Sciences, University of L'Aquila, 67100 L'Aquila, Italy; g Clinical Biochemistry Department, Lozano Blesa University Hospital, Instituto de Investigación Sanitaria de Aragón, Zaragoza, Spain; h Department of Obstetrics and Gynecology, Arnaud de Villeneuve Hospital, Montpellier, France; i College of Medicine, University of Ibadan, Ibadan, Nigeria; j University College Hospital, Ibadan, Nigeria; k Department of Obstetrics & Gynecology, Medical Faculty, Universitas Airlangga, Surabaya, Indonesia; l Soetomo General Academic Hospital, Surabaya, Indonesia; m Division Neonatología, Hospital Materno Infantil Ramón Sarda, Buenos Aires, Argentina; n Department of Obstetrics and Gynaecology, Faculty of Clinical Sciences, College of Medical Sciences, Gombe State University, Gombe, Nigeria; o Department of Obstetrics and Gynecology, Bordeaux University Hospital, Bordeaux, France; p Department of Obstetrics and Gynecology, Keio University School of Medicine, Tokyo, Japan; q Department of Obstetrics and Gynecology, The Jikei University School of Medicine, Tokyo, Japan; r Department of Paediatrics & Child Health, The Aga Khan University Hospital, Karachi, Pakistan; s School of Public Health, University of California, Berkeley, CA, USA; t Department of Pediatrics, General Hospital Borka Taleski, Prilep, Republic of North Macedonia; u Departments of Obstetrics and Gynecology and of Global Health, University of Washington, Seattle, WA, USA; v Obstetrics and Gynecology Unit, IRCCS Istituto Giannina Gaslini, Genova, Italy; w Obstetrics and Gynaecology, University of Brescia, Italy; x Elizabeth Garrett Anderson Institute for Women's Health, University College London, London, UK; y Fetal Medicine Unit, University College London Hospitals NHS Foundation Trust, London, UK; z Department of Woman Child and Neonate, Fondazione IRCCS Ca' Granda Ospedale Maggiore Policlinico, Obstetrics Unit, Milan, Italy; aa Hôpital Universitaire Necker-Enfants Malades, AP-HP, Université de Paris, France; ab Hôpitaux Universitaires de Genève, Département de la Femme, de l'Enfant et de l'Adolescent, Geneva, Switzerland; ac Instituto Nacional de Perinatología Isidro Espinosa de los Reyes, Mexico City, Mexico; ad Maternal and Child Department, Hospital Nacional Profesor Alejandro Posadas, Buenos Aires, Argentina; ae Obstetrics Department, Hospital Universitari Vall d’Hebron, Barcelona Hospital Campus, Barcelona, Spain; af Department of BioMedical and Clinical Sciences, Ospedale Luigi Sacco University Hospital, University of Milan, Milan, Italy; ag Department of Clinical and Community Sciences, University of Milan, Milan, Italy; ah S.C. Obstetrics 2U, Sant’Anna Hospital, AOU Città della Salute e della Scienza di Torino, Italy; ai Sanatorio Otamendi, Ciudad de Buenos Aires, Argentina; aj Servicio de Neonatología del Departamento Materno Infantil, Hospital Universitario Austral, Buenos Aires, Argentina; ak St George's University Hospitals NHS Foundation Trust, London, UK; al Tufts Medical Center, Boston, MA, USA; am Universidade Federal do Maranhão, São Luís, Brazil; an University of Calabar Teaching Hospital, Calabar, Nigeria; ao Africa Center of Excellence for Population Health and Policy, Nigeria; ap Ann and Robert H Laurie Children's Hospital of Chicago, IL, USA; aq Ascension–Resurrection Medical Center, Chicago, IL, USA; ar Centro Hospitalario Pereira Rossell, Montevideo, Uruguay; as Clínica y Maternidad Suizo Argentina, Argentina; at Departamento de Pediatria, Faculdade Medicina Universidade Federal de Minas Gerais, Belo Horizonte, Brazil; au Department of Obstetrics and Gynaecology, Abubakar Tafawa Balewa University Teaching Hospital, Bauchi, Nigeria; av Department of Obstetrics and Gynaecology, Muhammad Abdullahi Wase Teaching Hospital, Kano State, Nigeria; aw Department of Obstetrics and Gynecology, Koc University Hospital, Istanbul, Turkey; ax Division of Maternal-Fetal Medicine and Division of Critical Care Medicine, Brigham and Women's Hospital, Harvard Medical School, Boston, MA, USA; ay Fr. Thomas Alan Rooney Memorial Hospital, Asankragwa, Ghana; az Holy Family Hospital, Nkawkaw, Ghana; ba Hospital de Moron, Moron, Provincia de Buenos Aires, Argentina; bb Hospital Julio C Perrando, Resistencia, Chaco, Argentina; bc Hospital Regional Lic. Adolfo López Mateos ISSSTE, Mexico City, Mexico; bd Mainland Hospital Yaba Lagos, Nigeria; be National Medical Research Center for Obstetrics, Gynecology & Perinatology, Moscow, Russia; bf Nutrition and Dietetics, College of Health Sciences, University of Sharjah, Sharjah, United Arab Emirates; bg Obstetrics and Gynecology Division- Hadassah Medical Center Faculty of Medicine, Hebrew University of Jerusalem, Israel; bh Servicio de Neonatologia, Hospital Italiano de Buenos Aires, Instituto Universitario Hospital Italiano, Buenos Aires, Argentina; bi University of Nottingham Medical School, Nottingham, UK; bj Translational Health Science and Technology Institute, Faridabad, India; bk Tropical Medicine and Infectious Diseases Department, Tanta University, Tanta, Egypt; bl Universidad de Buenos Aires, Buenos Aires, Argentina; bm Universidad de Moron, Moron, Argentina; bn Universidad Nacional de Córdoba, Argentina; bo Center for Global Child Health, Hospital for Sick Children, Toronto, Canada

**Keywords:** Preeclampsia, COVID-19, SARS-CoV-2, Vaccine, Pregnancy

## Abstract

**Background:**

We tested whether COVID-19 vaccination affects the risk of preeclampsia (PE) given the well-documented association between COVID-19 and PE, and their overlapping risk factors and pathophysiological pathways.

**Methods:**

We analysed individual level data from pregnant women prospectively enrolled from 18 countries in two consecutive cohorts between 2020 and 2022 during the COVID-19 pandemic using identical methodology. Pregnant women were recruited either with a COVID-19 diagnosis or as concomitant, consecutive, non-diagnosed controls from the same hospitals. Following vaccine availability, vaccination status was documented to define a vaccine-exposed subgroup. Multivariable logistic regression models assessed the odds of PE adjusting for confounders and cohort as a proxy for viral strain, stratifying by pre-existing morbidities and SARS-CoV-2 infection. Survival analyses estimated PE incidence according to vaccination status and pre-existing morbidities.

**Findings:**

Of 6527 pregnant women, 2166 (33.2%) were diagnosed with COVID-19 and 3753 (57.5%) were unvaccinated. Of the 2774 vaccinated women, 1795 (64.7%) received mRNA vaccines; 848 (30.6%) received the initial regimen plus a booster dose, of whom 66.6% received a booster with an mRNA vaccine. We confirmed an independent association between COVID-19 and PE (aOR: 1.45; 95% CI: 1.15–1.84), particularly in unvaccinated women (aOR: 1.78; 95% CI: 1.31–2.42). Overall, after adjusting for confounders, any vaccination gave a protective effect against PE during the index pregnancy (aOR: 0.85; 95% CI: 0.65–1.10), that was stronger with a booster dose (aOR: 0.67; 95% CI: 0.45–0.99). Among women with pre-existing morbidities who received a booster dose the odds were reduced by 58% (aOR: 0.42; 95% CI: 0.20–0.87) – an effect mainly observed in women diagnosed with COVID-19. Adjustment for study site and cohort year did not alter the magnitude of the effect. Vaccination amongst women who received a booster dose was also associated with decreased odds of maternal (aOR: 0.68; 95% CI: 0.55–0.83) and perinatal (aOR: 0.71; 95% CI: 0.54–0.95) morbidity and mortality, and preterm birth (aOR: 0.67; 95% CI: 0.53–0.85).

**Interpretation:**

COVID-19 vaccination with a booster reduces the odds of PE by 30% approaching 60% reduction among women with pre-existing morbidities.

**Funding:**

The original INTERCOVID study was supported in Oxford by the COVID-19 Research Response Fund from the University of Oxford (Ref 0009083).

**Research in context**

**Evidence before this study**

Recent research has demonstrated a positive association between COVID-19 and PE with an increased risk of more than 60% in infected vs. non-infected pregnancies, according to a comprehensive meta-analysis. Viral infections are linked to PE through placental dysfunction, syncytiotrophoblast stress and inflammation. SARS-CoV-2 infection typically produces a cytokine storm with direct vascular endothelial damage, leading to microvascular injury, thrombosis, placental dysfunction and hypoxia. Moreover, dysregulation of the angiotensin converting enzyme 2 leads to vasoconstriction, hypertension, placental insufficiency and PE.

**Added value of this study**

This study addresses key knowledge gaps regarding: 1) the potential preventive effect of COVID-19 vaccination on the risk of PE; 2) the impact of shared maternal risk factors that may confound the association between COVID-19 and PE, and 3) the possible role of viral infection, immune system activation and their interplay in the aetiology of PE.

We confirmed the association between COVID-19 and PE, showing a 45% increased risk in the cohort across the pandemic and 78% among unvaccinated women after multivariable adjustment (aOR: 1.45; 95% CI: 1.15–1.84) and 1.78; 95% CI: 1.31–2.42, respectively). COVID-19 vaccination with a booster dose reduced PE risk by 33% in the overall cohort and by 58% among women with pre-existing morbidities (aOR: 0.67; 95% CI: 0.45–0.99, and 0.42; 95% CI: 0.20–0.87 respectively). Although the PE odds reduction provided by vaccination remained significant after adjusting for SARS-CoV-2 infection, it was greater among women diagnosed with COVID-19.

**Implications of all the available evidence**

Firstly, the well-established benefits of COVID-19 vaccination status during pregnancy are extended beyond disease severity, to the potential reduction of PE, a condition of uncertain aetiology. Therefore, efforts to vaccinate pregnant women should be strengthened, with particular emphasis on those with pre-existing morbidities. Secondly, a coordinated programme to understand PE aetiology better should focus on how the immune system interacts with infections in general, alone and in combination, with vaccination.

## Introduction

Previous evidence has suggested an association between maternal infection and preeclampsia (PE), supported by recent data showing that SARS-CoV-2 infection is significantly associated with an increased risk of PE during the COVID-19 pandemic.^1^, ^2^, ^3^ There is also a consistent overlap between COVID-19 and PE in terms of major risk factors, clinical outcomes, and underlying pathophysiological pathways.^4^ Concurrently, extensive research has confirmed the effectiveness and safety of COVID-19 vaccination^5^, ^6^, ^7^, ^8^, ^9^ in reducing the risk of disease severity, preterm birth, perinatal mortality, and adverse pregnancy outcomes, thereby supporting recommendations for vaccination before or during pregnancy.^3^^,^^10^ However, whether vaccination may also reduce the risk of PE, given the shared endothelial, inflammatory, and vascular dysfunction mechanisms between COVID-19 and PE, remains uncertain.

Understanding this potential relationship is clinically important. If vaccination mitigates the risk of PE, this will not only reinforce current vaccination strategies in pregnancy but also provide insight into shared pathogenic mechanisms between infection-related inflammation and hypertensive disorders of pregnancy. Such findings may also contribute to the broader understanding of PE pathophysiology beyond COVID-19.

Hence, the next logical step was to evaluate whether COVID-19 vaccination during pregnancy reduces the prevalence of PE, considering overall vaccine exposure, timing of vaccination, and pre-existing maternal health conditions. We aimed prospectively to 1) confirm the association between PE and COVID-19 diagnosis across the pandemic, and 2) investigate the independent impact of COVID-19 vaccination on the prevalence of PE according to vaccine dose, maternal comorbidities, and COVID-19 diagnosis.

## Methods

### Study design

This is an individual level combination of the two INTERCOVID prospective cohorts, involving 40 hospitals in 18 countries (Argentina, Brazil, Egypt, France, Indonesia, Israel, Italy, Japan, Mexico, Nigeria, North Macedonia, Pakistan, Spain, Switzerland, Turkey, UK, Uruguay, and USA). These two cohorts were studied, by the same research team with the primary focus on COVID-19 during pregnancy, from the start of the pandemic, using the same standardised protocols, data management and data collection strategy. Participating hospitals are part of the Oxford Maternal and Perinatal Health Institute's (OMPHI) global research network and deliver routine care to thousands of women and newborns annually using standardised protocols. Hospitals were chosen to maximise rapid enrolment of both diagnosed and non-diagnosed pregnant women, not to represent the broader population. Data management and analyses were conducted by the original statistical unit across the whole period.

Patients were recruited from March 2, 2020, to November 2, 2020 (INTERCOVID 2020) and from November 27, 2021, to June 30, 2022 (INTERCOVID 2022). The flow of patient selection and recruitment is presented in Supplemental Figure S1. The study is presented in agreement with the STrengthening the Reporting of OBservational studies in Epidemiology (STROBE) guidelines.^11^ A detailed description of the protocols and procedures is available at https://intergrowth21.tghn.org/intercovid/.

### Participants

The diagnostic criteria for both a COVID-19 diagnosis and non-diagnosis have been described in detail previously.^2^^,^^3^ Participants were enrolled at any time during pregnancy or childbirth and most diagnosed cases had laboratory evidence of SARS-CoV-2 infection by real time PCR or rapid test. Two unmatched, consecutive, unaffected women, at the same level of care (if possible), were recruited after each case, at each study site, serving as the control group. All live and stillborn singleton or multiple births, including those with congenital anomalies, were included. Maternal and neonatal outcomes were recorded up to hospital discharge. Additional information on inclusion and exclusion criteria can be found in the two original publications.^2^^,^^3^ The studies were approved by the Oxford Tropical Research Ethics Committee (OxTREC), ref no 526-20, and local ethics boards, with no impact on clinical care. Informed consent was obtained per local regulations, unless waived. The studies followed the Declaration of Helsinki and Good Clinical Practice, and the full protocol, including laboratory methods, has previously been published.^2^

### Procedures

Individual patient data from the INTERCOVID 2020 and 2022 cohorts were aggregated to create a merged database in which the study period was considered as indicator variable. During recruitment, screening occurred at any hospital admission, including for delivery, as well as when COVID-19 testing was performed based on symptoms, exposure, occupation, or risk status per local guidelines. To ensure consistency in the present analysis, we applied the same procedures, documentation, and data management system as our previous analyses.^1^, ^2^, ^3^^,^^10^^,^^12^^,^^13^

Gestational age was assessed using INTERGROWTH-21st standards^14^ or the best available obstetric data (crown rump length <14 weeks’ gestation and other ultrasound or clinical data). Newborn size was assessed using INTERGROWTH-21st standards for gestational age and sex^15^ or very preterm reference charts.^16^

Vaccination information was retrieved from verbal or medical reports, primary medical files, vaccination cards, vaccination registries and all other documentation systems and recorded immediately when vaccines were available in the study sites with type of vaccine (mRNA vs. non-mRNA), dates of vaccinations and completion with a booster dose. Vaccines were categorised as mRNA (Moderna mRNA-1273 or Pfizer-BioNTech BNT162b2) or non-mRNA (inactivated virus or viral vector types).

### Vaccination status definitions

Women were classified as “unvaccinated” (no doses or unknown status), “vaccinated” (completely or partially as per each vaccine company's protocol) or “boosted” (three doses or two of Ad26.COV2.S). Anyone who received a vaccine or booster during pregnancy was considered vaccinated or boosted for the analysis, regardless of pre-pregnancy status. In the analyses, completely and partially vaccinated were merged as “any vaccination”.

### Outcomes

The primary outcome of the study was PE. The diagnosis (obtained from pregnancy, antepartum admission to any health institution and delivery records) was defined as^1^: 1) blood pressure ≥140/90 mm Hg or an increase of at least 30 mm Hg systolic or 15 mm Hg diastolic from baseline, recorded on at least two occasions 6 or more hours apart, accompanied by proteinuria, occurring after 20 weeks’ gestation in a previously normotensive pregnancy; 2) blood pressure ≥160 mm Hg systolic or ≥110 mm Hg diastolic recorded on two occasions at least 4 h but no more than 168 h apart, or a single elevated measurement followed by immediate antihypertensive treatment, with either scenario accompanied by proteinuria; 3) eclampsia, characterised by convulsions or coma not attributable to other cerebral conditions, occurring in a patient with signs and symptoms of preeclampsia; or 4) haemolysis, elevated liver enzymes, and low platelet count (HELLP) syndrome.

Secondary outcomes were the: 1) Maternal morbidity and mortality index (MMMI) included at least one complication during pregnancy (pregnancy-induced hypertension, preeclampsia, eclampsia, haemolysis, elevated liver enzymes, vaginal bleeding, or low platelet count), preterm delivery, infection requiring antibiotics, maternal death, admission to an intensive care unit (ICU), or referral to higher dependency care. 2) Severe perinatal morbidity and mortality index (SPMNI) included bronchopulmonary dysplasia, hypoxic-ischaemic encephalopathy, sepsis, anaemia requiring transfusion, patent ductus arteriosus, intraventricular haemorrhage, necrotising enterocolitis, or retinopathy of prematurity, intrauterine or neonatal death, or a stay in the neonatal ICU of ≥7 days.

As a secondary objective, to further assess the safety of vaccination, we used logistic regression models to assess preterm delivery, MMMI and SPMMI as the outcomes with vaccination status (vaccinated any, booster dose) compared to unvaccinated adjusting for the same set of confounders included for PE.

### Pre-existing maternal morbidities

For the stratified analysis, and to be consistent with our previous publications, we considered pre-existing maternal morbidities as: diabetes mellitus, thyroid or any other endocrine disorders; cardiac disease; hypertension; chronic respiratory disease; kidney disease; or tuberculosis.^3^ Detailed definitions, standardised across study sites, are available at https://intergrowth21.com.

### Statistical methods

Our analytical strategy should be considered as a one-stage model for merging data from cohorts of individual pregnant women across time following the same study protocols. Comparisons between vaccinated and unvaccinated participants were made based on the “intention-to-treat” concept, i.e., if vaccinated, a pregnant woman remained in that group regardless of any COVID-19 diagnosis or any other clinical or pregnancy-related issue. We followed a pre-specified analysis for the main hypothesis and imputed missing data in sensitivity analyses. The sample size was justified *a priori* for both of our previous studies; therefore, no specific justification was required for this analysis as it represents a summary of the dataset reassessing PE as the primary outcome.

We enrolled 2242 and 4618 women during INTERCOVID-2020 and INTERCOVID-2022, respectively. We excluded women (n = 333) with missing data on overweight/obese, preeclampsia, previous pregnancies, tobacco use or pre-existing morbidities from our primary analyses. Thus, a total of 6527 women were included in this analysis (INTERCOVID-2020 = 2139; INTERCOVID-2022 = 4388), of whom 3753 (57.5%) were unvaccinated and 2774 (42.5%) vaccinated. Of the women who were vaccinated, 1926 (69.4%) received one or two doses, as per specific protocols and 848 (30.6%) received a booster dose. A COVID-19 diagnosis during pregnancy was given to 2166/6527 (33.2%) women and 330/6527 (5.1%) women were diagnosed with PE (Supplemental Figure S1).

Baseline characteristics, pregnancy history, pre-existing morbidities, and infections and treatments during pregnancy were described comparing: 1) unvaccinated women with women vaccinated with and without a booster dose, and 2) INTERCOVID 2020 and INTERCOVID 2022 cohorts (percentage or mean ± SD).

As our primary analyses, we used individual multivariable logistic regression models with PE as the outcome (dependent variable) to estimate the odds ratio (OR) and 95% confidence intervals (95% CI). We selected potential confounders *a priori* that were considered on the causal pathway using a directed acyclic graph (Supplemental Figure S2), described in more detail previously.^1^^,^^3^

We evaluated the association of PE with COVID-19 diagnosis during pregnancy compared to no diagnosis as the reference group adjusting for maternal age, tobacco use, previous pregnancies, overweight/obese, INTERCOVID 2020 or INTERCOVID 2022 cohort, antenatal aspirin prophylaxis, non-steroidal anti-inflammatories (NSAIDs), history of cardiac disease, hypertension, kidney disease and diabetes, and study site. We also evaluated the association among the 3753 unvaccinated women.

We estimated the odds of PE based on COVID-19 vaccination status (vaccinated any or booster dose) and timing of booster vaccination (vaccinated before pregnancy, or during 1st, 2nd or 3rd trimester), with unvaccinated as the reference group. In these models, we adjusted for maternal age, tobacco use, previous pregnancies, overweight or obese, antenatal aspirin prophylaxis, NSAIDs, history of cardiac disease, hypertension, kidney disease and diabetes. In secondary analyses, we evaluated the relationship between PE and vaccination status stratified by COVID-19 diagnosis while adjusting for these same variables. We also estimated the odds of PE with vaccination status among only those with pre-existing morbidities (n = 1142) adjusting for maternal age, tobacco use, previous pregnancies, overweight/obese, antenatal aspirin prophylaxis, and NSAIDs. Finally, we used a single multivariable logistic regression model to estimate adjusted odds ratios (aORs) of PE for all exposures and confounders included in the previous models (except for an interaction term for COVID-19 positive) to provide an overall assessment of the adjusted odds of each potential predictor variable. We adjusted also for year of cohort and included a category for partial vaccination in models.

We adjusted for COVID-19 diagnosis, focusing on the question of the protective effect of vaccination on PE. An additional question is whether such a protective effect is mediated by a false negative COVID-19 diagnosis, i.e., some women may have been less infected, tested negative for COVID-19, and thereby assigned to the non-diagnosed group. Alternatively, vaccination may reduce COVID-19 complications among COVID-19 positive women, including PE.

We used Kaplan–Meier estimates to graph the failure (incidence of PE) in relation to gestational age at birth. This was done to compare unvaccinated, vaccinated or booster dose in the whole cohort visually and in the sub-group with pre-existing morbidities. We tested the equality of the failure functions, using the log-rank test across vaccine groups after assessing the proportional hazards assumptions, and did exploratory analysis assessing the type of booster dose (mRNA vs. non-mRNA).

We then conducted sensitivity analyses exploring whether exclusion due to missing data influenced our results. We also treated each country in sensitivity analyses as if they were separate studies in a meta-analysis. We imputed missing values and assumed that if PE, pre-existing morbidities, previous pregnancies, tobacco use and overweight/obese were not reported they were not diagnosed.

Statistical significance for all main comparisons was considered as p < 0.05 and p < 0.1 for interactions. All analyses were performed with STATA 17.0.

### Role of the funding source

The funding organization had no involvement in the design and conduct of the study; collection, management, analysis, and interpretation of the data; preparation, review, or approval of the manuscript, and decision to submit the manuscript for publication. The views expressed herein are those of the authors and not necessarily those of the NHS, the NIHR the Department of Health or any of the other funders.

## Results

### COVID-19 diagnosis and preeclampsia

Baseline characteristics, pregnancy history, pre-existing morbidities, and infections and treatments during pregnancy according to INTERCOVID cohort are provided in Supplemental Table S1. Although values were similar, women enrolled in the INTERCOVID-2022 cohort were slightly older, heavier, less likely to work outside the home, have a university education, smoke and drink alcohol during pregnancy. Information about timing of SARS-CoV-2 infection during pregnancy, most likely variant, vaccine type and symptom severity is available in the original publications.^2^^,^^3^ However, for the two cohorts, the mean (±SD) gestational age at COVID-19 diagnosis was 33.8 ± 7.8 weeks. Among the controls, 3897/4361 (89.2%) tested negative for SARS-CoV-2 infection.

Table 1 presents the unadjusted and adjusted results for the association between COVID-19 diagnosis and PE. Similar results to the unadjusted analysis were obtained with the adjusted analysis: aOR 1.45 (95% CI 1.15–1.84) in the full population, reaching aORs of 1.78 (95% CI 1.31–2.42) among unvaccinated women (Table 1). The rate of severe COVID-19 was 0.1% in the booster dose group vs. 2.7% among the unvaccinated women (p < 0.001). Only six women reported COVID-19 diagnosis before pregnancy, three of whom developed PE. We did not adjust for COVID-19 prior to pregnancy in the complete analysis.

**Table 1:** The association between COVID-19 diagnosis and preeclampsia (PE).

Model	Status	N	PE cases	Unadjusted		Adjusted^a^	
				OR (95% CI)	p value	OR (95% CI)	p value
All pregnancies (n = 6527)	COVID−	4361	192 (4.4)	Ref.		Ref.	
	COVID+	2166	138 (6.4)	1.48 (1.18, 1.85)	0.001	1.45 (1.15, 1.84)	0.002
Unvaccinated (n = 3753)	COVID−	2471	104 (4.2)	Ref.		Ref.	
	COVID+	1282	94 (7.3)	1.80 (1.35, 2.40)	0.00006	1.78 (1.31, 2.42)	0.0003

a Adjusted for maternal age, tobacco use, previous pregnancies, overweight or obese, INTERCOVID 2020 or INTERCOVID 2022 cohort, antenatal aspirin prophylaxis, non-steroidal anti-inflammatories, history of cardiac disease, hypertension, kidney disease and diabetes, and study site.

### COVID-19 vaccination and preeclampsia

The baseline characteristics among women according to vaccination status are shown in Table 2. In general, vaccinated women with a booster dose were taller, more educated, more likely to be married or cohabiting, and worked less outside the home; none received more than one booster. They had fewer non-SARS-CoV-2 infections and took more multivitamins and aspirin, factors that are both protective for PE and associated with increased vaccination patterns. However, these women were older, smoked less, drank more, had lower parity, and took fewer calcium supplements, factors that could affect risk for PE.

**Table 2:** Baseline characteristics among pregnant women according to vaccination status.

	Unvaccinated (n = 3753)	Vaccinated, no booster (n = 1926)	Vaccinated with Booster (n = 848)
Demographic and socioeconomic characteristics			
Maternal age (years)	30.3 ± 6.1	31.3 ± 6.0	33.7 ± 5.0
Maternal height (cm)	161.7 ± 7.5	162.5 ± 7.9	163.6 ± 6.9
Maternal pre-pregnancy or 1st trimester weight (kg)	67.5 ± 16.2	68.7 ± 18.6	66.2 ± 14.2
Body mass index (kg/m^2^)	25.8 ± 5.8	26.0 ± 6.5	24.8 ± 5.2
Married or cohabitating (%)	87.1	87.3	90.6
University education (%)	30.3	34.9	59.5
Worked outside the home (%)	48.6	38.1	17.0
Smoker during index pregnancy (%)	5.3	6.7	3.7
Alcohol use during pregnancy (%)	1.8	3.5	4.4
Obstetric history			
Previous pregnancy (%)	69.5	68.0	60.3
Previous miscarriage (%)	30.8	30.5	28.1
Previous birth (%)	57.9	55.2	46.4
Previous baby <2.5 kg or >4.5 kg (%)	7.8	6.8	4.9
Previous baby <37 weeks' gestation (%)	5.9	5.9	4.7
Previous stillbirth or neonatal death (%)	3.3	3.0	2.6
Previous adverse pregnancy outcome (%)	36.9	36.0	32.3
Maternal pre-existing morbidities			
Diabetes (%)	2.2	3.1	2.0
Thyroid or other endocrine disease (%)	9.4	10.4	13.9
Cardiac disease (%)	1.5	2.2	2.1
Hypertension (%)	2.5	3.3	2.7
Chronic respiratory disease (%)	1.3	0.7	1.8
Kidney disease (%)	0.8	1.1	0.5
Malaria (%)	0.9	0.0	0.0
Tuberculosis (%)	0.2	0.4	0.6
≥2 of the above conditions (%)	1.8	2.7	2.7
Infections and treatments during pregnancy			
Urinary tract infection (%)	5.5	7.4	4.0
Pyelonephritis (%)	0.4	0.4	0.0
Other infection requiring antibiotics (%)	3.5	5.0	2.7
Aspirin (%)	9.7	14.4	15.8
Antibiotics (except for PROM) (%)	13.0	12.9	9.8
Antibiotics for PROM (%)	6.6	7.3	5.4
Non-steroidal anti-inflammatories (%)	1.6	3.1	5.2
Insulin (%)	4.5	5.9	3.9
Steroids for PROM (%)	7.7	7.6	5.8
Calcium supplement (%)	20.0	12.5	8.4
Multivitamins (%)	49.9	46.9	57.5

PROM, premature rupture of membranes.

The mean (±SD) gestational age at the time of the last vaccine dose was 20.2 ± 13.7 weeks and the time between the last vaccine dose and delivery was 18.5 ± 13.2 weeks. Only six women had a pre-pregnancy COVID-19 diagnosis and 9.4% of women had their last vaccine dose before the start of pregnancy.

Among vaccinated women (n = 2774), 1796 (64.7%) received an mRNA vaccine and 921 (33.2%) a non-mRNA vaccine (we could not confirm the type of vaccine in 57 women). Among women having a booster dose (n = 848), 565 received an mRNA vaccine (66.6%) and 280 (33.0%) a non-mRNA vaccine. Finally, among women with pre-existing morbidities that were vaccinated (n = 522), 364 (69.7%) received an mRNA vaccine; among those having a booster dose (n = 173), 129 (74.6%) received an mRNA vaccine and 43 (24.8%) a non-mRNA vaccine.

The prevalence of PE for all women showed a non-statistically significant reduction from 5.3% among the unvaccinated to 4.0% among those with a booster dose (OR: 0.75; 95% CI: 0.52–1.09); this effect was larger among women with pre-existing morbidities from 10.5% to 5.2% for unvaccinated and booster dose, respectively (OR: 0.47; 95% CI: 0.23–0.96) (Table 3). In the adjusted model, there was a large protective effect for booster dose (aOR: 0.67 (95% CI: 0.45–0.99) for the association between COVID-19 vaccination and PE in the total population. When only women with pre-existing morbidities were considered, the odds of PE, adjusted for possible confounding variables, were substantially reduced by 58% (aOR: 0.42; 95% CI: 0.20–0.87) for the booster dose (Table 3).

**Table 3:** The association between COVID-19 vaccination status and preeclampsia (PE).

Status	N	PE cases	Unadjusted		Adjusted	
			OR (95% CI)	p value	OR (95% CI)	p value
All pregnancies (n = 6527)						
Vaccination status^a^						
Unvaccinated	3753	198 (5.3)	Ref		Ref.	
Vaccinated (any)	1926	98 (5.1)	0.96 (0.75, 1.23)	0.76	0.85 (0.65, 1.10)	0.21
Booster	848	34 (4.0)	0.75 (0.52, 1.09)	0.13	0.67 (0.45, 0.99)	0.04
Pregnancies with pre-existing morbidities^b^ (n = 1142)						
Vaccination status^c^						
Unvaccinated	620	65 (10.5)	Ref.		Ref.	
Vaccinated (any)	349	37 (10.6)	1.01 (0.66, 1.55)	0.95	0.94 (0.61, 1.46)	0.79
Booster	173	9 (5.2)	0.47 (0.23, 0.96)	0.04	0.42 (0.20, 0.87)	0.02

a Models adjusted for maternal age, tobacco use, previous pregnancies, overweight or obese, antenatal aspirin prophylaxis, non-steroidal anti-inflammatories, and history of cardiac disease, hypertension, kidney disease and diabetes.

b Pre-existing health conditions included as a binary variable (yes or no) any pre-existing maternal morbidities (including diabetes, thyroid, and other endocrine disorders; cardiac disease; hypertension; chronic respiratory disease; kidney disease; or tuberculosis.

c Models adjusted for maternal age, tobacco use, previous pregnancies, antenatal aspirin prophylaxis, and non-steroidal anti-inflammatories.

Although further adjustment for study site and cohort year produced wider CI, it did not change the magnitude of the reduced odds of PE among women with a booster dose during pregnancy. Despite some comparisons not reaching statistical significance, there was a very consistent pattern of a protective effect of vaccination on PE, with a similar substantial effect among women with pre-existing morbidities (Supplemental Table S2).

Exploring the timing of booster dose in relation to the odds of PE seems to suggest vaccination's protective effect was predominant among those vaccinated during the 2nd or 3rd trimester (Supplemental Table S3).

**Fig. 1:**
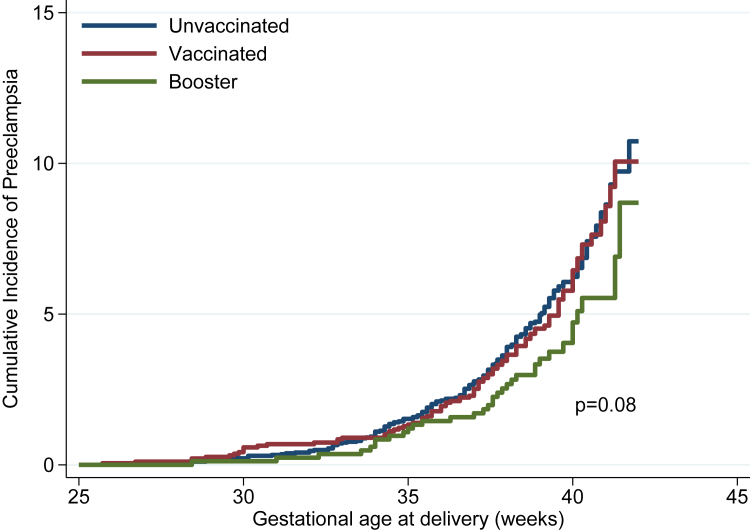
Kaplan–Meier curves of preeclampsia cumulative incidence by vaccination status (n = 6527).

**Fig. 2:**
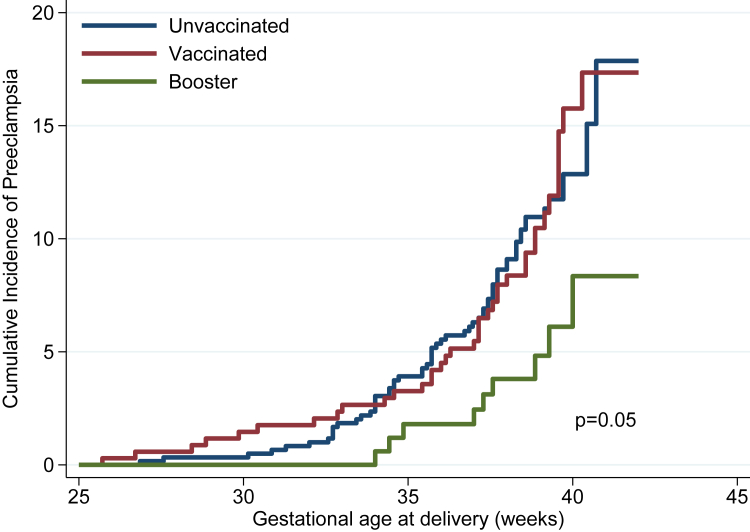
Kaplan–Meier curves of preeclampsia cumulative incidence by vaccination status among pregnancies with pre-existing health conditions (n = 1142).

The Kaplan–Meier curves visually present the effect of the boosted women with a lower cumulative incidence of PE (log rank test: p value = 0.08), starting at about 35 weeks’ gestation, as compared with the unvaccinated and any vaccination without a booster dose (Fig. 1). This effect was more evident among women with pre-existing morbidities, as shown in Table 3 (log rank test: p value = 0.05) (Fig. 2). Stratified analyses according to the type of vaccine (mRNA vs. non-mRNA) used as a booster dose for all women or those with pre-existing morbidities did not demonstrate any differential patterns between these two types of vaccine.

Table 4 shows the unadjusted and adjusted associations between vaccination status and PE according to COVID-19 diagnosis status, as compared to unvaccinated. Among COVID-19 diagnosed women in the adjusted analysis, any vaccination (aOR: 0.58; 95% CI: 0.37–0.89) was protective against PE and booster dose showed a non-significant protection (aOR: 0.62; 95% CI: 0.31–1.23). Among women not diagnosed with COVID-19 the effect among women with a booster dose (aOR: 0.74; 95% CI: 0.45–1.20) did not reach statistical significance for either vaccination group. However, we acknowledge the limitations of over-stratification with very small numbers of PE per cell, producing wide CI of the OR.

**Table 4:** The association between COVID-19 vaccination status and preeclampsia (PE) stratified according to COVID-19 diagnosis, all pregnancies in the study.

All pregnancies (n = 6527)	Vaccination status	COVID−			COVID+			p value_interact
		N	PE cases	OR (95% CI)	N	PE cases	OR (95% CI)	
Unadjusted models	Unvaccinated	2471	104 (4.2)	Ref.	1282	94 (7.3)	Ref.	
	Vaccinated (any)	1268	65 (5.1)	1.23 (0.90, 1.69)	658	33 (5.0)	0.67 (0.44, 1.00)#	0.02
	Booster dose	622	23 (3.7)	0.87 (0.55, 1.38)	226	11 (4.9)	0.65 (0.34, 1.23)	0.45
Adjusted models^a^	Unvaccinated	2479	104 (4.2)	Ref.	1285	94 (7.3)	Ref.	
	Vaccinated (any)	1268	65 (5.1)	1.08 (0.78, 1.50)	658	33 (5.0)	0.58 (0.37, 0.89)∗	0.02
	Booster dose	622	23 (3.7)	0.74 (0.45, 1.20)	226	11 (4.9)	0.62 (0.31, 1.23)	0.57

a Models adjusted for maternal age, tobacco use, previous pregnancies, overweight or obese, antenatal aspirin prophylaxis, non-steroidal anti-inflammatories, and history of cardiac disease, hypertension, kidney disease and diabetes. ∗P < 0.05; #P < 0.1.

Overall, results were very similar in sensitivity analyses with imputation of missing values as negative response for both PE and confounders. Unfortunately, the number of PE cases was too small to assess differences in the type of booster vaccine in the stratified analysis.

A summary of the independent effect of COVID-19 vaccination on PE, along with the independent effect of the co-variants that we adjusted for, is presented in multivariable logistic regression models in Table 5. (Table 5 includes only co-variants that remained significant at p < 0.05.) In these models, we found a non-statistically significant (p = 0.06) reduction of the odds for PE of 32% after a booster dose in the total population and a 56% reduction in PE odds among women with pre-existing morbidities after a booster dose (p = 0.03; upper CI below the null hypothesis) when compared with unvaccinated women (Table 5). These summary models confirm the results presented in Tables 3 and 4. Supplemental Table S4 shows the full results including non-significant predictors.

**Table 5:** Results from multivariable logistic regression analysis of the association between COVID-19 vaccination status and preeclampsia diagnosis with potential predictors of risk identified in the study population.

Predictor	Adjusted^a^ OR (95% CI)	p value
All pregnancies (n = 6527)		
COVID-19 vaccination (any)	0.85 (0.65, 1.10)	0.21
Booster dose	0.68 (0.46, 1.01)	0.06
COVID-19 diagnosis positive	1.38 (1.09, 1.74)	0.007
Overweight	1.57 (1.19, 2.08)	0.001
Obesity	2.15 (1.61, 2.86)	1.7 × 10^−7^
Previous pregnancies	0.62 (0.49, 0.80)	2.0 × 10^−4^
History of hypertension	5.53 (3.78, 8.08)	1.1 × 10^−18^
Pregnancies with pre-existing morbidities^b^ (n = 1142)	c	
Vaccinated (any)	0.95 (0.61, 1.48)	0.82
Booster dose	0.44 (0.21, 0.92)	0.03
Smoking during pregnancy	2.16 (1.07, 4.39)	0.03
Previous pregnancies	0.62 (0.40, 0.96)	0.03

a Model includes, in addition to the presented variables, maternal age, smoking during pregnancy, history of cardiac disease, history of kidney disease, history of diabetes, antenatal aspirin prophylaxis or non-steroidal anti-inflammatory use during pregnancy.

b Pre-existing morbidities include at least one of the following: diabetes, thyroid, and other endocrine disorders; cardiac disease; hypertension, chronic respiratory disease; kidney disease; or tuberculosis.

c Model includes, in addition to the presented variables, maternal age, antenatal aspirin prophylaxis or non-steroidal anti-inflammatory use during pregnancy.

Independent of vaccination status, confounding variables were associated with an increased odds of PE: history of hypertension, obesity/overweight, and COVID-19 diagnosis among all women, and smoking during pregnancy among women with pre-existing morbidities (Table 5).

Finally, logistic regression models with other indicators of severe adverse maternal and neonatal outcomes show that women with any vaccination had an independent protective effect for these outcomes (Table 6). Amongst women that had the booster dose, the protective effect for preterm birth was aOR: 0.67; 95% CI: 0.53–0.85, for maternal morbidity and mortality index was aOR: 0.68; 95% CI: 0.55–0.83, and for severe perinatal morbidity and mortality index was aOR: 0.71; 95% CI: 0.54–0.95. The protective effect of vaccination for these outcomes was largest among COVID-19 diagnosed women (Table 6).

**Table 6:** Associations between COVID-19 vaccination status and adverse maternal and neonatal outcomes.

Condition	Vaccination status	N	Cases	Unadjusted (all) OR (95% CI)	Adjusted (all) OR (95% CI)^a^	COVID– OR (95% CI)	COVID+ OR (95% CI)	p value_int
Preterm delivery	Unvaccinated	3744	601 (16.1)	Ref.	Ref.	Ref.	Ref.	
	Vaccinated (any)	1921	248 (12.9)	0.78 (0.66, 0.91)∗	0.75 (0.64, 0.88)∗	0.84 (0.68, 1.03)#	0.63 (0.48, 0.82)∗	0.07
	Booster dose	848	94 (11.1)	0.65 (0.52, 0.82)∗	0.67 (0.53, 0.85)∗	0.87 (0.66, 1.14)	0.36 (0.21, 0.60)∗	0.002
Maternal morbidity and mortality index^b^	Unvaccinated	3753	829 (22.1)	Ref.	Ref.	Ref.	Ref.	
	Vaccinated (any)	1926	370 (19.2)	0.84 (0.73, 0.96)∗	0.79 (0.68, 0.90)∗	0.99 (0.83, 1.18)	0.53 (0.41, 0.67)∗	<0.001
	Booster dose	848	144 (17.0)	0.72 (0.59, 0.88)∗	0.68 (0.55, 0.83)∗	0.79 (0.62, 1.02)#	0.53 (0.36, 0.78)∗	0.001
Severe perinatal morbidity and mortality index^c^	Unvaccinated	3833	391 (10.2)	Ref.	Ref.	Ref.	Ref.	
	Vaccinated (any)	1962	171 (8.7)	0.84 (0.70, 1.02)#	0.81 (0.67, 0.99)∗	0.99 (0.77, 1.27)	0.61 (0.45, 0.83)∗	0.01
	Booster dose	867	65 (7.5)	0.71 (0.54, 0.94)∗	0.71 (0.54, 0.95)∗	0.83 (0.59, 1.17)	0.61 (0.37, 1.00)∗	0.32

∗P < 0.05; ^#^P < 0.1.

a Models adjusted for maternal age, tobacco use, previous pregnancies, overweight or obese, antenatal aspirin prophylaxis, non-steroidal anti-inflammatories, and history of cardiac disease, hypertension, kidney disease and diabetes.

b Maternal morbidity and mortality index includes at least one complication during pregnancy (pregnancy-induced hypertension, preeclampsia, eclampsia, haemolysis, elevated liver enzymes, vaginal bleeding, or low platelet count), preterm delivery, infection requiring antibiotics, maternal death, admission to an intensive care unit (ICU), or referral to higher dependency care.

c Severe perinatal morbidity and mortality index includes any of the morbidities listed in the Severe neonatal morbidity index, intrauterine or neonatal death, or a stay in the neonatal ICU of 7 days or longer.

### Sensitivity analysis

A sensitivity analysis exploring whether exclusion due to missing data influenced our results was carried out treating each country as if they were separate studies in a meta-analysis. The pooled estimated ORs (95% CI) were very similar for booster dose, but with wider confidence intervals, as expected. For the adjusted model of vaccination status and PE, the OR was 0.45 (0.13, 1.61) in the multivariable logistic regression model among those with pre-existing conditions.

## Discussion

This analysis confirms the association between SARS-CoV-2 infection and PE with an increase of 45% risk during the pandemic and 78% among unvaccinated women during the same period, adjusted for major risk factors. Importantly, for a disease of uncertain aetiology such as PE, we have demonstrated that vaccination with a booster dose, after adjusting for a comprehensive set of possible confounding variables including COVID-19 diagnosis, study site and year of the pandemic, reduced the odds of PE by over 30% in the complete cohort and by about 58% among women with pre-existing morbidities. This protective effect was consistently extended to other severe maternal and perinatal morbidity and mortality indicators.

Experimental and clinical studies have suggested that viral infections during pregnancy could disrupt several key steps in the shared pathway leading to the clinical manifestations of PE. This process may be initiated through mechanisms related to dysfunctional placentation with perturbation of trophoblast invasion with deep placentation, syncytiotrophoblast stress due to vascular damage in placental vessels, and maternal systemic inflammation.^17^, ^18^, ^19^, ^20^

Several viruses have been associated with PE risk including SARS-CoV-2, HIV, HHV6, parvovirus B19, HSV, HPV, AAV-2, CMV, hepatitis viruses, influenza and ZIKAV, each with their own different putative mechanisms.^20^, ^21^, ^22^, ^23^ Specifically, COVID-19 typically produces a cytokine storm paired with direct vascular endothelial damage by the virus, leading to microvascular injury, thrombosis, placental dysfunction and hypoxia.^24^ Moreover, dysregulation of the angiotensin converting enzyme 2 resulting from different mechanisms such as viral entry into host cells, inflammatory responses, increased angiotensin II activity, endothelial damage and hypoxia, can lead to vasoconstriction, hypertension, placental insufficiency, and eventually PE.^25^, ^26^, ^27^, ^28^

Although, the aetiology of PE remains uncertain, immune system changes and vascular dysfunction are consistently reported to play a role in its pathophysiology, interacting with infective agents.^24^ To address such complexity, a unified model integrating these factors with the different phenotypes of PE has been proposed.^24^^,^^25^

To elucidate the specific mechanisms responsible for the observed results is beyond the scope of the present report although some exploratory analyses were done. The PE reduction observed could be partially mediated by a direct effect of vaccines reducing overall COVID-19 diagnoses (the rate of COVID-19 diagnosis was 27% in the booster dose group vs. 34% among the unvaccinated women) or the prevention of its more severe forms (the rate of severe COVID-19 was 0.1% in the booster dose group vs. 2.7% among the unvaccinated women). Also, vaccination's protective effect against PE was strongest among COVID-19 diagnosed women; however, we have demonstrated that the protective effect of vaccination remains after adjusting for COVID-19 diagnosis.

Alternatively, vaccination may enhance the immune system through prevention of other infections or reduced immune-mediated vascular maternal or placental damage. There is evidence that live-attenuated vaccines such as Bacille Calmette-Guerin, measles or oral polio vaccine may reduce all-cause mortality beyond their specific targets, perhaps via heterologous immunity, i.e., antigen cross-reactivity through molecular mimicry.^28^ Such immune system enhancement may arise from broad immune responses including cytokine signalling, bystander lymphocyte activation, or innate immune memory via macrophages and NK cells, driven by epigenetic reprogramming.^28^

The available literature is neither comprehensive nor consistent. A population-based study from Australia of 270,000 women with 3700 PE cases assessed the rate of PE after vaccines for influenza, pertussis or both. A small reduction in the rate of PE was found among vaccinated vs. unvaccinated women (influenza vaccine: 2.7% vs. 2.5%; aRR: 0.94; 0.87–1.00; pertussis vaccine: 2.7% vs. 2.5%; aRR: 0.93; 0.87–0.99; both vaccines vs. neither: 2.7 vs. 2.5; aRR: 0.94; 0.87–1.00), with higher statistical significance achieved when restricting the analysis to primiparous women (influenza vaccine: 4.1% vs. 3.7%; aRR: 0.89; 0.82–0.98; pertussis vaccine: 2.7% vs. 2.5%; aRR: 0.93; 0.87–0.99; both vaccines vs. neither: 3.8% vs. 4.1%; aRR: 0.90; 0.82–0.98). The authors proposed that maternal vaccination may be associated with improved pregnancy outcomes via immunomodulatory effects, reducing harmful systemic inflammation.^28^ Another study showed that vaccination against Respiratory Syncytial Virus during pregnancy had no statistically significant effect on rates of PE and eclampsia in the vaccinated group (PE: vaccine 2.4% vs. placebo 2.7%; eclampsia: vaccine 0.2% vs. placebo 0.4%).^29^

A matched cohort study found no overall difference in PE rates between COVID-19 vaccinated and unvaccinated groups but noted slightly higher PE risk with 1st trimester vaccination, suggesting a time-dependent effect likely influenced by more high-risk women being vaccinated earlier.^30^ Finally, vaccinated pregnant women with hypertensive disorders who later became infected with SARS-CoV-2 had similar maternal, perinatal outcomes and PE biomarker levels to uninfected women.^31^

Our prospective cohorts had adequate sample size based on the original hypotheses; however, the current results are from secondary analyses, limiting the statistical power for sub-group stratification particularly when assessing vaccine types or covariates related to possible mediating mechanisms (e.g., COVID-19 before pregnancy). Selection bias to vaccination status is also an area of concern for the evaluation of causality in a non-randomised intervention. We made numerous comparisons increasing the chance of finding false positive results; however, the effect estimates were large and consistent across our different analyses increasing confidence in our findings. Although there was some risk of misclassification of COVID-19, especially early in the pandemic, this would likely attenuate the relationships and result in an underestimate of the effect.

We adjusted for known potential confounders in the analysis, although residual and unmeasured confounding remains a factor in the interpretation of the quantitative effect. Importantly, some confounders exert opposing influences, which may result in a net balancing effect, e.g., low-dose antenatal aspirin (a preventative measure to lower the risk of PE) was taken by 16% in the booster dose group vs. 10% in the unvaccinated group; conversely, women in the booster dose group were older, smoked less, and had lower parity – all factors that increase the risk of PE. While some risk factors (older age, lower parity) may counterbalance, these factors introduce residual confounding, potentially contributing to the observed protective effect of vaccination on PE. Lastly, we used the classical definition of PE and did not consider organ dysfunction in the absence of proteinuria.^32^^,^^33^ Hence, we could not distinguish classical PE from PE-like forms associated with COVID-19,^34^^,^^35^ as specific markers were not available in this large multi-country collaboration.

Strengths include: 1) across the pandemic period we have confirmed the association between COVID-19 and PE in a large, multinational, prospective cohort of pregnant women, specifically designed to study COVID-19 during pregnancy; 2) the preventive effects of vaccination are strong and consistent across sub-groups, both in the crude and adjusted analyses; 3) although the 95% CIs were not narrow in any sub-group, nevertheless above 50% prevention was seen among women with pre-existing morbidities, a magnitude seldom observed in any preventive strategies for PE, and 4) even among the general pregnant population prevention reached 30%, which was however not a statistically significant reduction.

We have confirmed the independent association between COVID-19 diagnosis and PE and documented a robust effect of COVID-19 vaccination with a booster dose for PE prevention, particularly among women with pre-existing morbidities, for whom vaccination plays the role of a leveller of the PE risk toward that of the general population. However, caution should be exercised because of the observational nature of the study, the risk of selection bias related to vaccination uptake, possible residual confounding, and the risk of overstating results without statistical significance with reduced power in stratified analyses.

## Contributors

All authors confirm that their full access to all the data in the study and accept responsibility to submit for publication. All co-authors conceptualised and designed the study. All co-authors participated in data collection. The data analysis was conducted by RG, JV and PIC. The writing of the original draft was done by JV, PIC, SHK, ATP and RG. The reviewing and editing was done by JV, PIC, SHK, ATP and RG.

JV, RG, SR and ATP take responsibility for the integrity of the data and the accuracy of the data analysis. All authors have seen and approved the final text. All authors had full access to all the data in the study, although not all authors will have accessed the totality of the data set; rather, they will have accessed their own study site data. All authors had final responsibility for the decision to submit for publication.

## Data sharing statement

The study protocol and all data collection forms are available to all at https://intergrowth21.com. The anonymised database is only accessible to designated personnel as part of a data sharing agreement.

## Declaration of interests

P.D. reports serving as a lecturer for Norgine, Exeltis, and Biocodex. M.F. declares a research grant from the Instituto de Salud Carlos III (CM22/00045). M.G. declares sitting on the executive board of Global Pregnancy Collaboration with no financial compensation and holding 0.01% of total company equity in ModalityDx. L.Se. reports receiving consulting fees from Ferring Pharmaceuticals, Organon, Bayer, GlaxoSmithKline and Pfizer and serving as a lecturer for Ferring Pharmaceuticals, Bayer, Norgine, Pfizer, GlaxoSmithKline and Organon.
