# Supplementary material for: COVID-19 vaccination status during pregnancy and preeclampsia risk: the pandemic-era cohort of the INTERCOVID consortium
Source: eClinicalMedicine. 2026 Feb 18;93:103785. doi: 10.1016/j.eclinm.2026.103785 (PMC13043319; doi:10.1016/j.eclinm.2026.103785)

**Supplemental Table 1.** Baseline characteristics according to INTERCOVID Cohort.

|  | INTERCOVID 2020  (n=2139) | INTERCOVID 2022  (n=4388) | p-value |
| --- | --- | --- | --- |
| Demographic and socioeconomic characteristics |  |  |  |
| Maternal age (years) | 30.2 ± 6.1 | 31.4 ± 6.0 | <0.01 |
| Maternal height (cm) | 161.1 ± 7.6 | 162.7 ± 7.5 | <0.01 |
| Maternal pre-pregnancy or 1^st^ trimester weight (kg) | 65.8 ± 15.6 | 68.6 ± 17.2 | <0.01 |
| Body mass index (kg/m^2^) | 25.3 ± 5.8 | 25.9 ± 6.0 | <0.01 |
| Married or cohabitating (%) | 88.4 | 87.3 | 0.18 |
| University education (%) | 32.3 | 37.0 | <0.01 |
| Worked outside the home (%) | 49.1 | 37.7 | <0.01 |
| Smoker during index pregnancy (%) | 3.7 | 6.3 | <0.01 |
| Alcohol use during pregnancy (%) | 1.9 | 3.0 | 0.02 |
|  |  |  |  |
| Obstetric history |  |  |  |
| Previous pregnancy (%) | 68.2 | 67.7 | 0.67 |
| Previous miscarriage (%) | 31.1 | 30.0 | 0.36 |
| Previous birth (%) | 56.5 | 55.2 | 0.33 |
| Previous baby <2.5 kg or >4.5 kg (%) | 8.3 | 6.6 | 0.02 |
| Previous baby <37 weeks gestation (%) | 6.4 | 5.4 | 0.11 |
| Previous stillbirth or neonatal death (%) | 3.6 | 2.9 | 0.13 |
| Previous adverse pregnancy outcome (%) | 37.5 | 35.3 | 0.08 |
|  |  |  |  |
| Maternal pre-existing morbidities |  |  |  |
| Diabetes (%) | 2.5 | 2.4 | 0.88 |
| Thyroid or other endocrine disease (%) | 9.3 | 10.7 | 0.07 |
| Cardiac disease (%) | 1.5 | 1.9 | 0.16 |
| Hypertension (%) | 2.6 | 2.8 | 0.71 |
| Chronic respiratory disease (%) | 1.6 | 1.0 | 0.06 |
| Kidney disease (%) | 1.0 | 0.8 | 0.30 |
| Malaria (%) | 1.5 | 0.0 | <0.01 |
| Tuberculosis (%) | 0.3 | 0.3 | 0.68 |
| ≥2 of the above conditions (%) | 2.1 | 2.2 | 0.74 |
|  |  |  |  |
| Infections and treatments during pregnancy |  |  |  |
| Urinary tract infection (%) | 5.3 | 6.1 | 0.21 |
| Pyelonephritis (%) | 0.5 | 0.3 | 0.27 |
| Other infection requiring antibiotics (%) | 3.6 | 4.0 | 0.47 |
| Aspirin (%) | 9.7 | 12.9 | <0.01 |
| Antibiotics (except for PROM) (%) | 15.3 | 11.2 | <0.01 |
| Antibiotics for PROM (%) | 6.8 | 6.6 | 0.74 |
| Non-steroidal anti-inflammatories (%) | 1.6 | 2.9 | <0.01 |
| Insulin (%) | 4.0 | 5.2 | 0.04 |
| Steroids for PROM (%) | 7.5 | 7.4 | 0.95 |
| Calcium supplements (%) | 23.7 | 12.7 | <0.01 |
| Multivitamins (%) | 47.9 | 51.0 | 0.02 |
|  |  |  |  |
| Preeclampsia (%) | 5.8 | 4.7 | 0.07 |

PROM = premature rupture of membranes

**Supplemental Table 2.** The association between COVID-19 vaccination status and preeclampsia adjusting for site and cohort.

|  |  |  |  | **Unadjusted** | | **Adjusted** | | **Also adjust for study site** | | **Also adjust for cohort year** | |
| --- | --- | --- | --- | --- | --- | --- | --- | --- | --- | --- | --- |
| All pregnancies^a^ (n=6527) | **Status** | **N** | **Cases** | **OR (95% CI)** | **P**  **value** | **OR (95% CI)** | **P**  **value** | **OR (95% CI)** | **P**  **value** | **OR (95% CI)** | **P**  **value** |
| Vaccination status | Unvaccinated | 3753 | 198 (5.3) | Ref |  | Ref. |  | Ref |  |  |  |
|  | Vaccinated (any) | 1926 | 98 (5.1) | 0.96 (0.75, 1.23) | 0.76 | 0.85 (0.65, 1.10) | 0.21 | 0.83 (0.63, 1.10) | 0.19 | 0.80 (0.58, 1.12) | 0.19 |
|  | Booster dose | 848 | 34 (4.0) | 0.75 (0.52, 1.09) | 0.13 | 0.67 (0.45, 0.99) | 0.04 | 0.72 (0.47, 1.10) | 0.13 | 0.69 (0.43, 1.10) | 0.12 |
|  |  |  |  |  |  |  |  |  |  |  |  |
| Pregnancies with pre-existing morbidities^b^ (n=1142) |  |  |  |  |  |  |  |  |  |  |  |
| Vaccination status^c^ | Unvaccinated | 620 | 65 (10.5) | Ref. |  | Ref. |  | Ref. |  | Ref. |  |
|  | Vaccinated (any) | 349 | 37 (10.6) | 1.01 (0.66, 1.55) | 0.95 | 0.94 (0.61, 1.46) | 0.79 | 0.92 (0.55, 1.55) | 0.76 | 0.85 (0.45, 1.60) | 0.62 |
|  | Booster | 173 | 9 (5.2) | 0.47 (0.23, 0.96) | 0.04 | 0.42 (0.20, 0.87) | 0.02 | 0.45 (0.19, 1.08) | 0.07 | 0.42 (0.16, 1.07) | 0.07 |

^a^ Models adjusted for maternal age, tobacco use, previous pregnancies, overweight or obese, antenatal aspirin prophylaxis, non-steroidal anti-inflammatories, and history of cardiac disease, hypertension, kidney disease and diabetes.

^b^ Pre-existing health conditions included as a binary variable (yes or no) any pre-existing maternal morbidities (including diabetes, thyroid, and other endocrine disorders; cardiac disease; hypertension; chronic respiratory disease; kidney disease; or tuberculosis.

^c^ Models adjusted for maternal age, tobacco use, previous pregnancies, antenatal aspirin prophylaxis, and non-steroidal anti-inflammatories.

**Supplemental Table 3:** Results from multivariable logistic regression analysis of the association between COVID-19 vaccination status and preeclampsia diagnosis with potential predictors of risk identified in the study population for all predictors.

| Predictor | Adjusted OR (95% CI) | p-value |
| --- | --- | --- |
| All pregnancies (n=6527) |  |  |
| COVID-19 Vaccination (any) | 0.85 (0.65, 1.10) | 0.21 |
| Booster dose | 0.68 (0.46, 1.01) | 0.06 |
| COVID-19 diagnosis positive | 1.38 (1.09, 1.74) | 0.007 |
| Maternal age | 1.01 (0.99, 1.03) | 0.30 |
| Smoking during pregnancy | 1.30 (0.83, 2.01) | 0.25 |
| Overweight | 1.57 (1.19, 2.08) | 0.001 |
| Obesity | 2.15 (1.61, 2.86) | 1.7x10^-7^ |
| Previous pregnancies | 0.62 (0.49, 0.80) | 2.0x10^-4^ |
| History of cardiac disease | 1.65 (0.88, 3.08) | 0.12 |
| History of hypertension | 5.53 (3.78, 8.08) | 1.1x10^-18^ |
| History of kidney disease | 1.88 (0.80, 4.41) | 0.15 |
| History of diabetes | 1.41 (0.82, 2.42) | 0.21 |
| Aspirin use during pregnancy | 2.01 (1.51, 2.68) | 1.8x10^-6^ |
| NSAID use during pregnancy | 1.35 (0.72, 2.55) | 0.35 |
| Pregnancies with pre-existing morbidities^a^ (n=1142) |  |  |
| Vaccinated (any) | 0.95 (0.61, 1.48) | 0.82 |
| Booster dose | 0.44 (0.21, 0.92) | 0.03 |
| COVID-19 diagnosis positive | 1.33 (0.88, 2.02) | 0.18 |
| Maternal age | 0.99 (0.96, 1.03) | 0.73 |
| Smoking during pregnancy | 2.16 (1.06, 4.39) | 0.03 |
| Previous pregnancies | 0.62 (0.40, 0.96) | 0.03 |
| Aspirin use during pregnancy | 2.15 (1.40, 3.31) | 4.8x10^-4^ |
| NSAID use during pregnancy | 2.07 (0.85, 5.00) | 0.11 |

^a^ Pre-existing health conditions included as a binary variable (yes or no) any pre-existing maternal morbidities (including diabetes, thyroid, and other endocrine disorders; cardiac disease; hypertension, chronic respiratory disease; kidney disease; or tuberculosis.

**Supplemental Figure 1.** Enrollment flow chart for the combined INTERCOVID studies.


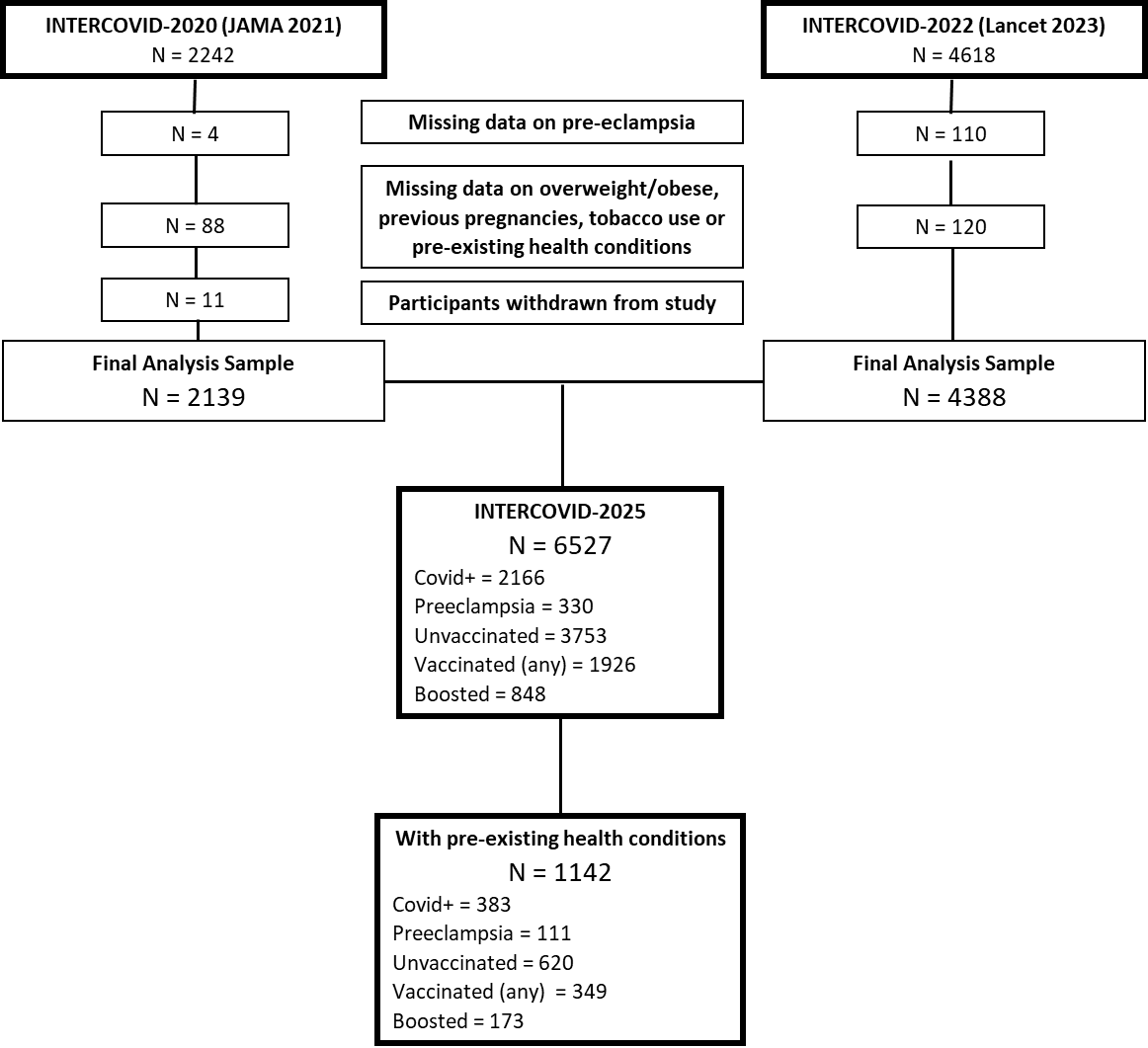


**Supplemental Figure 2.** Directed acyclic graph for the relationship between COVID-19 vaccination and preeclampsia.


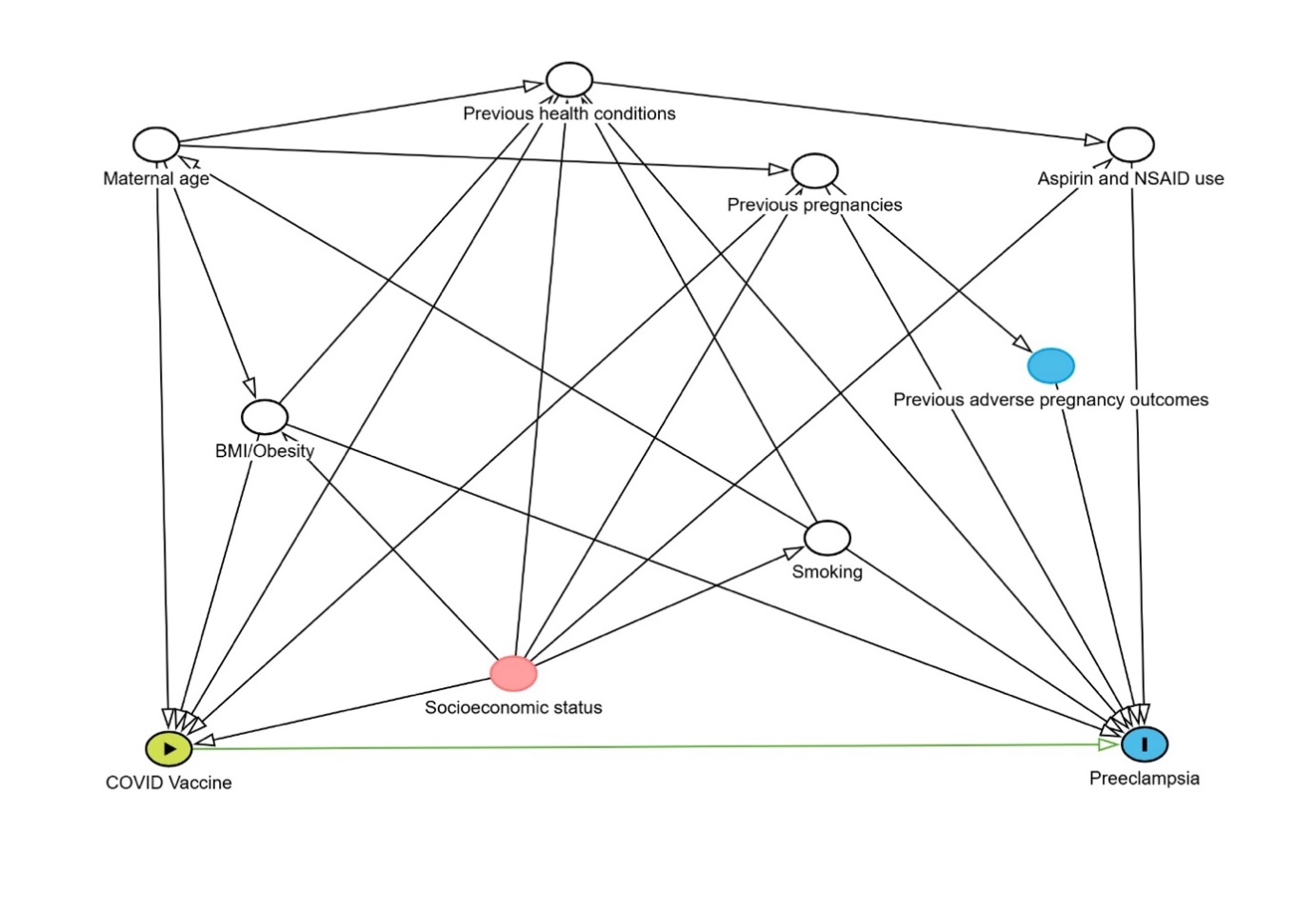

Supplement: Supplementary Tables 1–3 and Figures 1 and 2 [file mmc1.docx]
